# Solitary Brain Lesion: An Unexpected Presentation of Urachal Adenocarcinoma

**DOI:** 10.7759/cureus.97365

**Published:** 2025-11-20

**Authors:** Inês Q Dunões, Maria Baió, Francisco Trinca, Maria Inês Carvalhinho, Rui Dinis

**Affiliations:** 1 Medical Oncology, Unidade Local de Saúde do Alentejo Central, Évora, PRT; 2 Anatomical Pathology, Unidade Local de Saúde (ULS) de São José, Lisbon, PRT

**Keywords:** fluoropyrimidine-platinum chemotherapy, multimodal approach, solitary brain metastasis, urachal adenocarcinoma, whole-brain radiotherapy

## Abstract

Urachal adenocarcinoma (UA) is a rare and aggressive malignancy that typically presents at advanced stages and is associated with poor prognosis. We describe the case of a 63-year-old man with a history of UA treated with partial cystectomy seven years earlier, who remained under annual oncologic follow-up, with the last assessment nine months before the current episode. He presented with neurological symptoms initially suggestive of ischemic stroke. Brain magnetic resonance imaging (MRI) revealed a left cerebellar lesion, and due to its symptomatic nature and diagnostic uncertainty, surgical resection was performed. The biopsy showed metastatic adenocarcinoma, without definitive identification of the primary site. Subsequent staging identified only pulmonary lesions suggesting lung cancer; however, the diagnosis was made after a transthoracic needle biopsy, which confirmed UA in an advanced stage. The patient underwent whole-brain radiotherapy followed by systemic chemotherapy with fluoropyrimidine and platinum. Despite disease progression, he achieved prolonged disease control and preserved quality of life, living more than one year with an Eastern Cooperative Oncology Group (ECOG) performance status of 1 until the final weeks of life. This case shows that UA may present with late brain metastasis and supports a multidisciplinary treatment approach.

## Introduction

Urachal adenocarcinoma (UA) is a very rare and aggressive cancer, representing <1% of bladder cancers and approximately 0.01% of all adult malignancies [1,2]. These tumors arise from the embryologic remnants of the urachus at the bladder dome and are often diagnosed at advanced stages due to nonspecific symptoms [3]. 

For localized diseases, surgery remains the gold standard of therapy, most often partial or radical cystectomy with en bloc resection of the urachal ligament and umbilicus [3]. There is currently no standard protocol for adjuvant radiotherapy, as these tumors are generally radioresistant. Furthermore, neoadjuvant and adjuvant chemotherapy may be considered, especially in patients with a high number of lymph node involvement and in surgically unresectable patients, although its impact on survival is not well established [4].

Follow-up of UA typically involves regular clinical assessments, thoraco-abdominopelvic computed tomography (CT) scans, and sometimes monitoring of tumor markers, with imaging recommended every 6-12 months during the first 2-3 years and annually thereafter. Although the frequency of evaluations may be reduced after the first two years, surveillance is generally continued for at least 7-10 years. Given the limited literature, recurrence occurring beyond the initial two years is considered late, and ongoing long-term follow-up is advised to detect such late recurrences [5].

Prognosis is poor when distant metastases are present, with median survival ranging from 12 to 24 months [6]. The most frequent metastatic sites include the liver, lung, bone, and peritoneum, while brain metastases are rare [7,8], involving an especially challenging approach and a particularly worse prognosis.

Given the rarity of UA, standard chemotherapy regimens for metastatic disease have not been defined. However, fluoropyrimidine and platinum-based chemotherapy regimens are mostly used, extrapolated from gastrointestinal oncology protocols given histopathological similarities [4,9].

Recent studies have also shown that UA share molecular and histopathological characteristics with colorectal carcinomas, including microsatellite instability (MSH2, MSH6) and RAS and BRAF mutations, highlighting the potential role of targeted therapies and immunotherapy in the future [10-12].

## Case presentation

A 63-year-old man presented to the emergency department in June 2024 with dizziness, imbalance, and occipital headache with no respiratory or other systemic symptoms. He was an active smoker, with a 20 pack-year history, and had a past medical history of hypertension treated with olmesartan (20 mg), type 2 diabetes managed with metformin (1000 mg), and atrial fibrillation treated with rivaroxaban (20 mg).

His oncologic history included a UA diagnosed seven years earlier, treated with partial cystectomy and lymph node dissection in April 2017, followed by prolonged clinical and radiological surveillance. Abdominopelvic CT scans were performed every four months during the first two years, every six months in the subsequent years, and annually after five years of follow-up. Throughout this period, the patient remained asymptomatic, with an ECOG performance status of 0, and his last CT scan and clinical evaluation had been conducted nine months before the current presentation.

**Figure 1:**
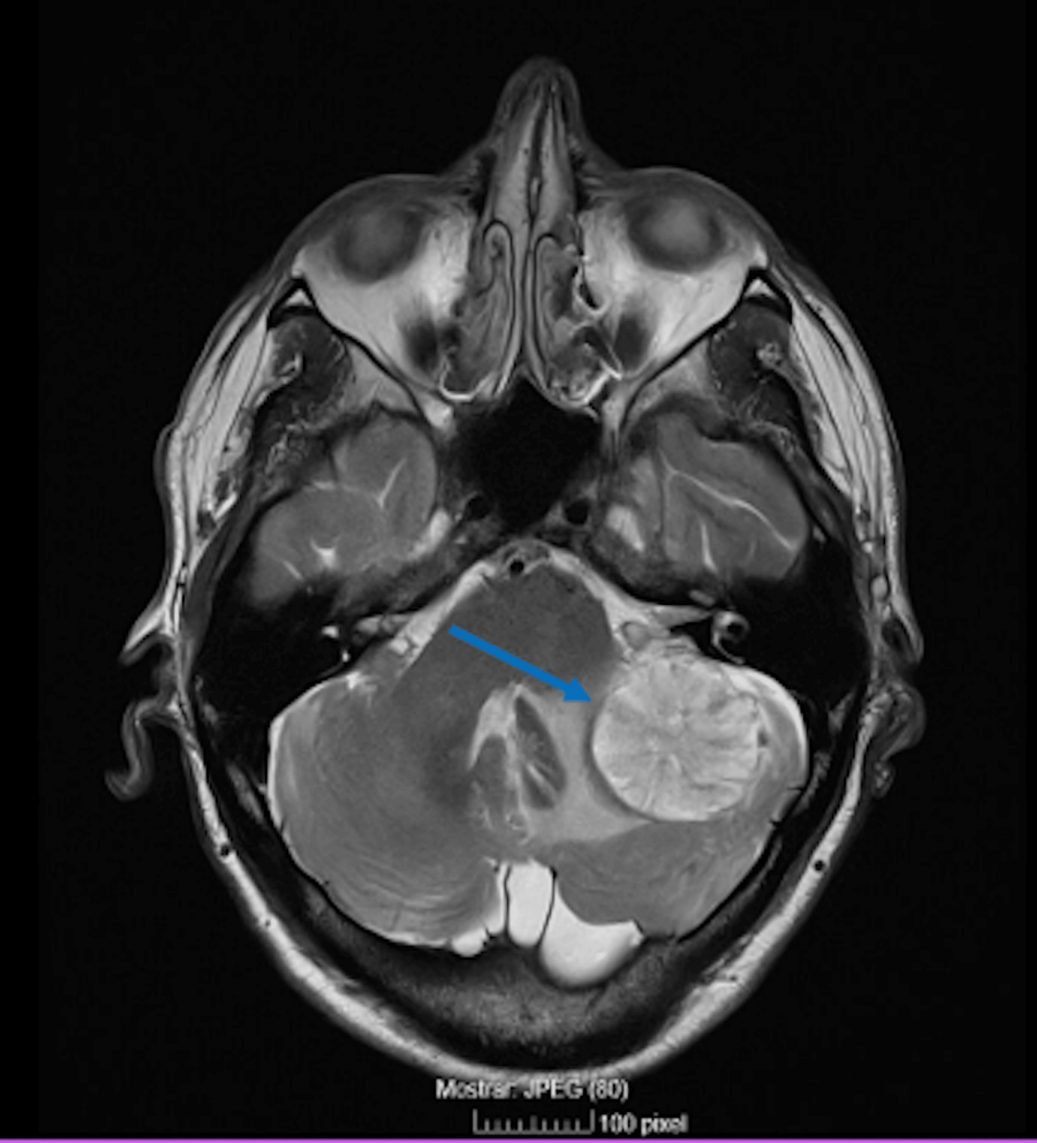
Brain MRI showing an intra-axial expansive lesion in the left posterior fossa measuring 37 × 37 × 35 mm

On admission, neurological examination revealed gait instability without focal deficits, and blood analysis was all normal. A non-contrast brain CT showed a left cerebellar cortico-subcortical hypodensity, initially interpreted as an ischemic stroke. Due to progression of symptoms, a brain magnetic resonance imaging (MRI) was performed, which revealed a 37 × 37 × 35 mm intra-axial lesion in the left posterior fossa with surrounding edema and mass effect (Figure 1). Considering the symptomatic nature of the lesion and diagnostic uncertainty, the patient started high-dose corticosteroids, and a craniotomy with resection of the brain lesion was performed.

Histopathological analysis demonstrated mucinous adenocarcinoma with signet-ring cells, raising differential diagnostic possibilities, including gastrointestinal, pulmonary, or urachal primary origin.

**Figure 2:**
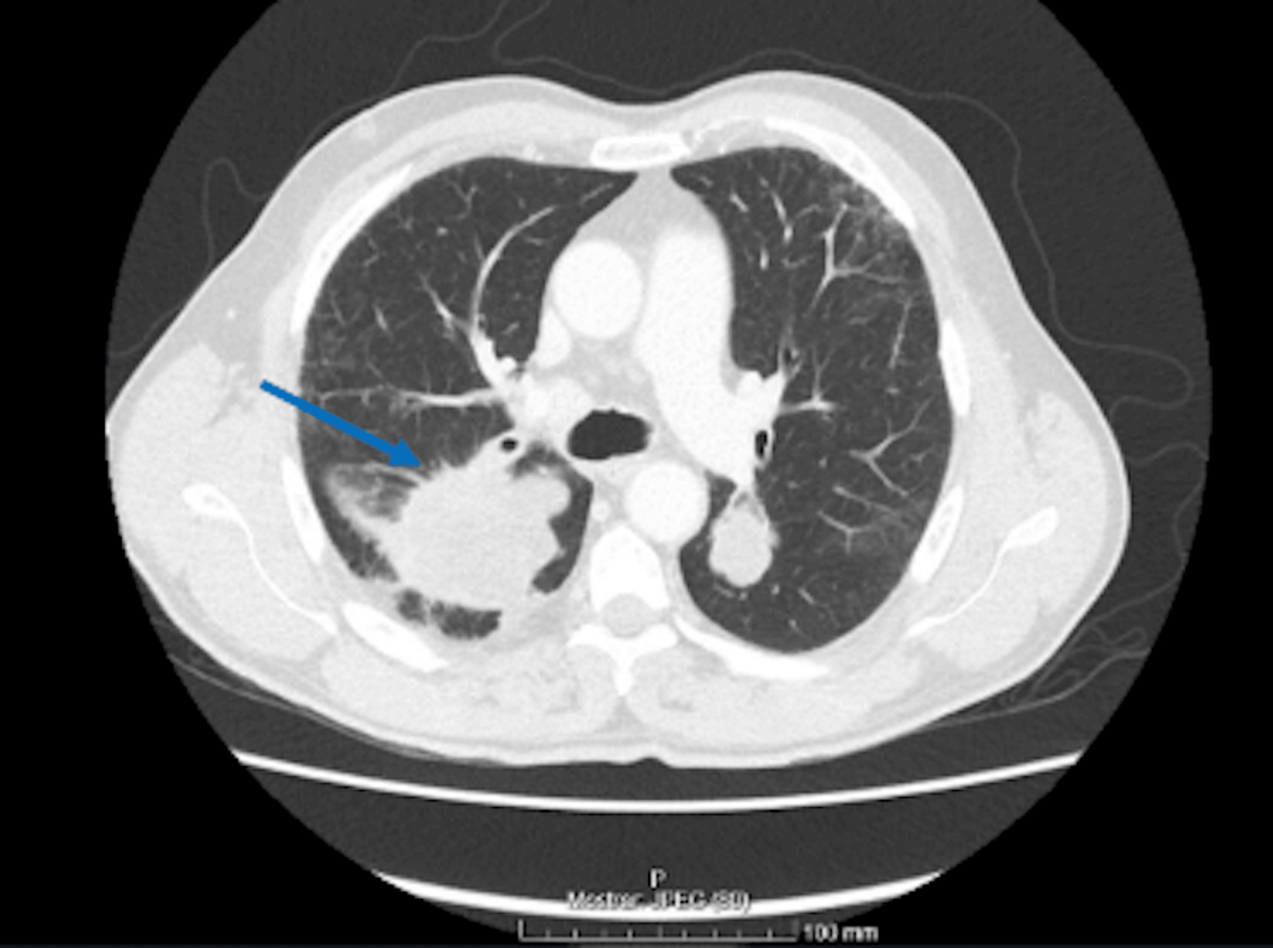
Abdominal, pelvic, and thoracic CT showing a mass in the right lung measuring 76 × 67 × 63 mm in the posterior segment of the upper lung lobe

Subsequent contrast-enhanced abdominal, pelvic, and thoracic CT revealed multiple lung nodules, the largest located in the right lung and measuring 76 × 67 × 63 mm in the posterior segment of the upper lobe, with extension into the apical segment of the lower lobe (Figure 2). Multiple lymphadenopathies were also noted in the tracheal, subcarinal, and right hilar regions.

Considering the patient’s oncologic history, a pelvic MRI was conducted to evaluate local recurrence, which was excluded. A positron emission tomography-computed tomography (PET-CT) performed in August 2024 to assess other possible sites of involvement revealed two hypermetabolic pulmonary masses: one in the left lower lobe (40 × 32 mm, SUVmax 7.3) and another in the right upper lobe (65 × 55 mm, SUVmax 11.7). Multiple hypermetabolic mediastinal and right hilar lymph nodes were also noted (SUVmax up to 7.4), with no other suspicious lesions identified.

**Figure 3:**
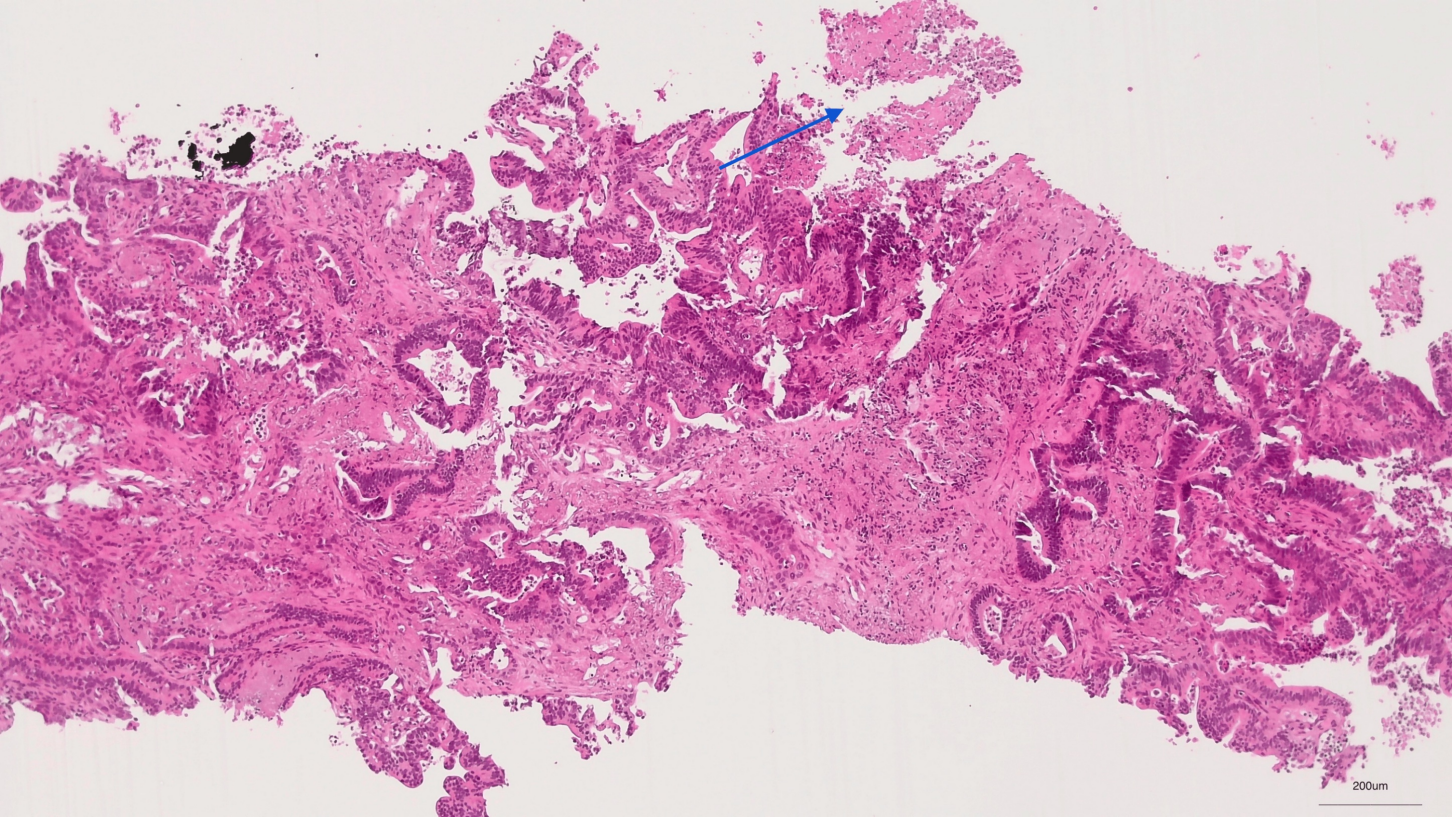
Extensively necrotic neoplasm, with tubular/acinar to cribriform architecture and composed of columnar cells with pseudostratified nuclei (H&E, 50x)

**Figure 4:**
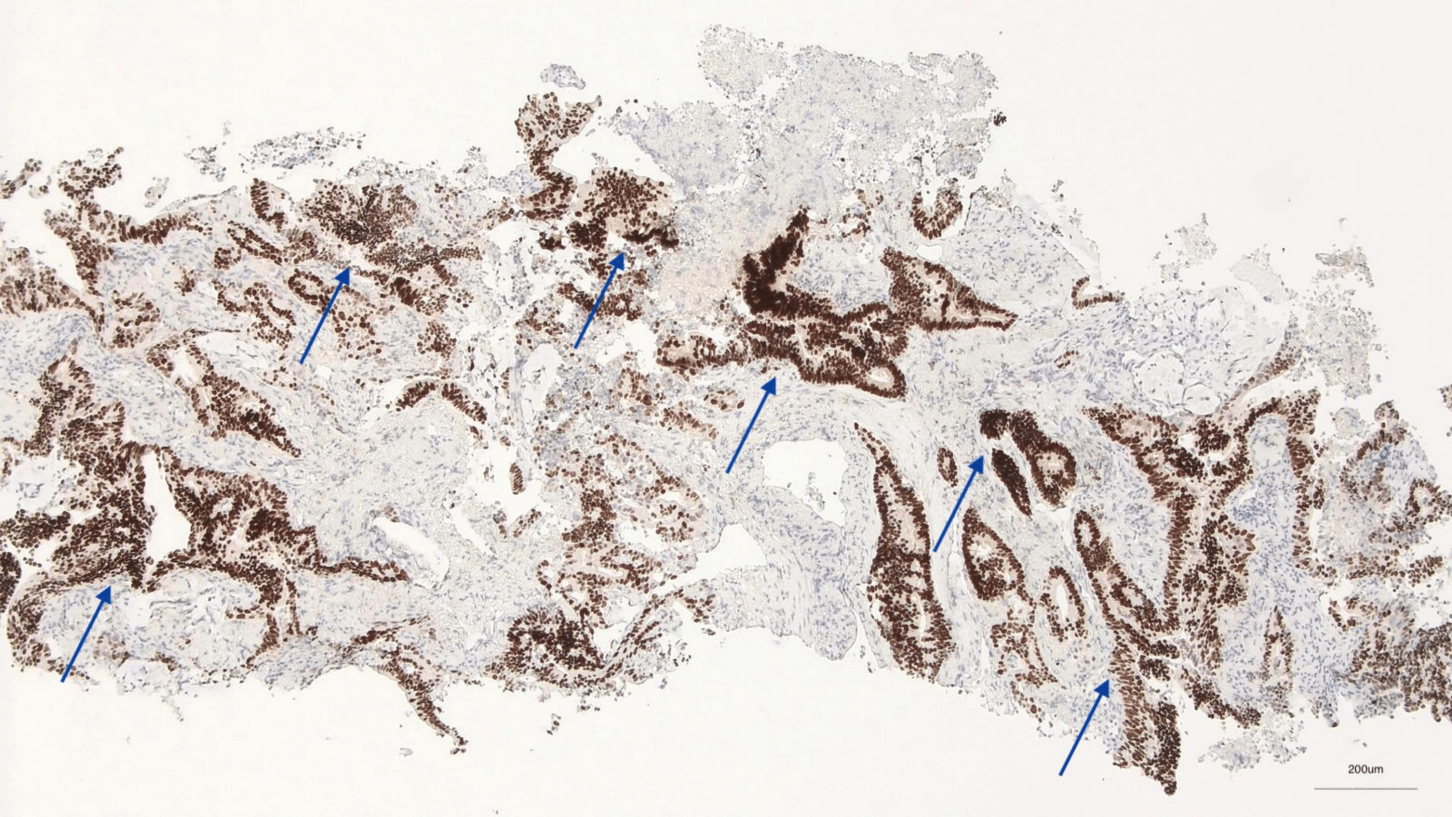
Nuclear staining of neoplastic cells (CDX2, 50x)

**Figure 5:**
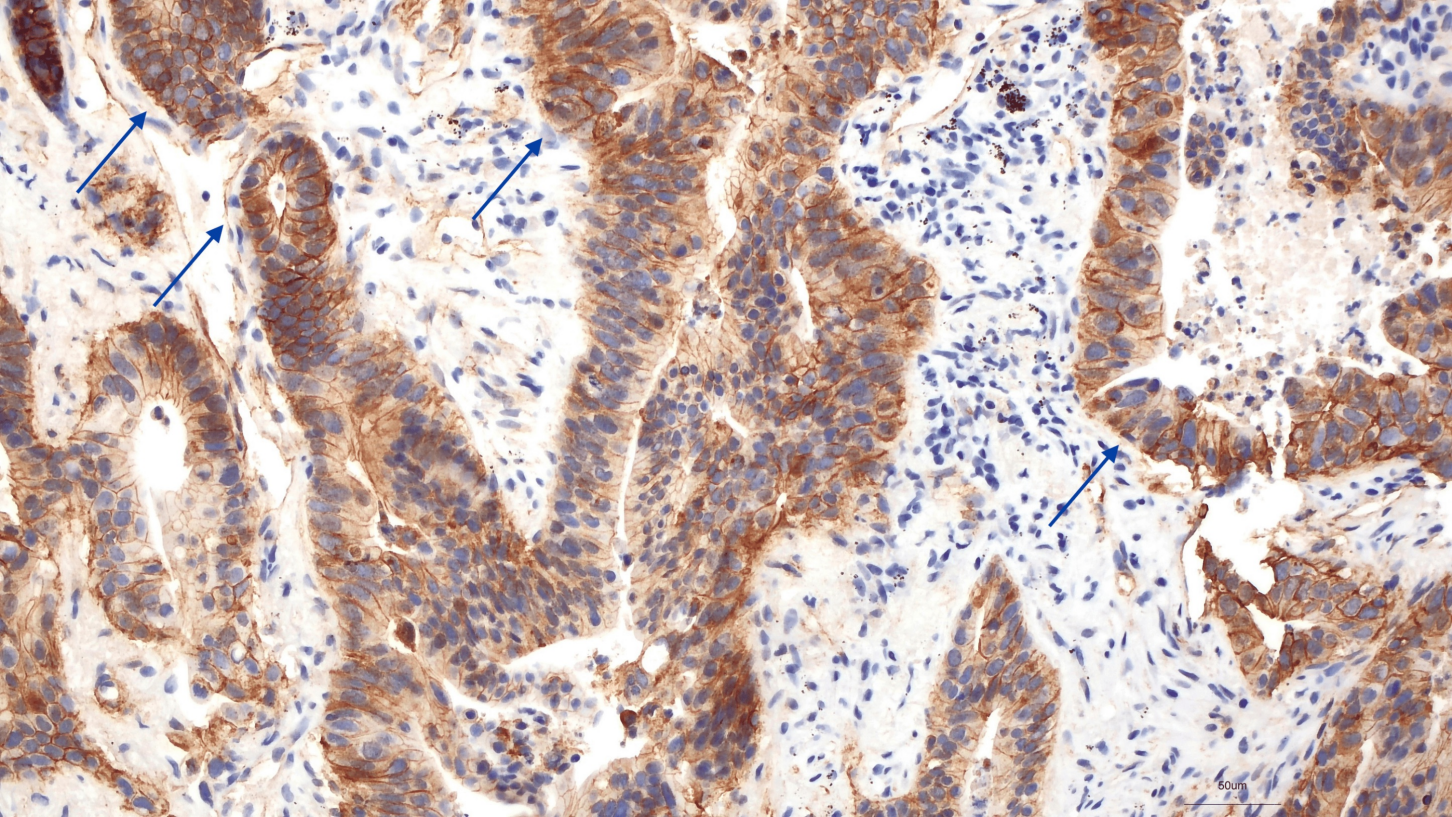
Absence of nuclear staining pattern (beta-catenin, 200x)

Given the suspicion of primary lung cancer, the patient underwent a transthoracic needle biopsy, which revealed an extensively necrotic neoplasm, with morphological and immunohistochemical features that, in the context of the patient’s clinical history, were consistent with metastatic adenocarcinoma of urachal origin (Figures 3-5).

The case was further discussed with the tumor board, and it was decided to initiate treatment with capecitabine (1000 mg/m^2^) twice a day and cisplatin (80 mg/m2) every 21 days in October 2024. Around the same time, the patient underwent whole-brain radiotherapy, with a total dose of 30 Gy delivered in 10 fractions (3 Gy per fraction).

In early 2025, after completing six cycles of cisplatin and capecitabine, abdominal, pelvic, and thoracic CT showed a partial response. However, due to poor treatment tolerance (nausea grade 3) and progressive decline in general condition, the regimen was switched to leucovorin, fluorouracil, and oxaliplatin (FOLFOX) every 14 days. 

The patient remained clinically stable with ECOG performance status 1, tolerating chemotherapy well and maintaining a good quality of life for several months. In August 2025, he presented to the emergency department with dyspnea. Abdominal, pelvic, and thoracic CT revealed marked disease progression, and the patient died shortly thereafter. Despite the aggressive nature of the disease, the patient lived for more than one year with metastatic UA, maintaining functional independence until the final weeks of life.

A summary of the main clinical events and respective dates is presented in Table 1.

**Table 1:** Summary of the main clinical events and corresponding dates

Date	Events
2017	The patient was diagnosed with localized urachal adenocarcinoma.
April 2017	Underwent partial cystectomy with lymph node dissection.
April 2017 to June 2024	Oncology follow-up: no changes in laboratory or imaging results, physical examination unremarkable, and no new symptoms reported.
June 2024 to August 2024	Presented to the emergency department with neurological symptoms. Brain CT: left cerebellar hypodensity (suspected ischemic stroke). Brain MRI: 37 × 37 × 35 mm left posterior fossa lesion with edema and mass effect. Craniotomy and resection: histopathology - mucinous adenocarcinoma with signet-ring cells but was inconclusive regarding the primary tumor origin. Chest/abdomen/pelvis CT: multiple lung nodules (largest 76 × 67 × 63 mm), mediastinal adenopathy. Pelvic MRI: no local recurrence. PET-CT: two hypermetabolic pulmonary masses, and multiple hypermetabolic mediastinal and right hilar lymph nodes were observed without other suspicious lesions.
September 2024	Transthoracic needle biopsy: metastatic urachal adenocarcinoma.
October 2024 to February 2025	Started chemotherapy: capecitabine + cisplatin and whole-brain radiotherapy 30 Gy/10 fractions.
February 2025 to July 2025	Switched to FOLFOX due to toxicity and clinical decline.
August 2025	Presented to the emergency department with dyspnea and CT scan showed marked disease progression. Patient deceased - survival with metastatic disease ~14 months, maintaining independence until final weeks.

## Discussion

In this patient, UA recurred after seven years of surveillance, presenting unusually as a solitary symptomatic cerebellar metastasis. The initial clinical suspicion of ischemic stroke highlights the diagnostic challenge, as neurological manifestations of urachal cancer are exceedingly rare.

At the time of recurrence, seven years after the initial diagnosis, the solitary symptomatic lesion was treated with surgical resection, providing local control and symptom relief. This approach is supported by previous reports suggesting that aggressive local therapy, including surgery or radiosurgery combined with radiotherapy, can improve local control [8,13,14].

At the same time, staging investigations identified multiple pulmonary metastases, and given the burden of disease, a multimodal strategy was adopted. Postoperative whole-brain radiotherapy (30 Gy/10 fractions) and fluoropyrimidine and platinum-based chemotherapy regimens provided radiological partial response and clinical stability.

In this case of late recurrent UA with metastases, the multimodal approach provided immediate neurological improvement after surgery, durable local control with radiotherapy, and systemic disease stabilization with chemotherapy. In addition, the patient maintained an ECOG performance status of 1 and preserved quality of life for many months, ultimately surviving more than one year with metastatic UA.

## Conclusions

Late recurrence in UA is a recognized phenomenon, reflecting the tumor’s indolent behavior and variable aggressiveness. Recurrences may occur several years after initial treatment, most commonly beyond two years, and this pattern justifies the need for prolonged clinical and radiological surveillance. Despite metastatic spread most frequently involving the liver, lungs, or peritoneum, this case shows that late brain metastases can occur, even seven years after initial treatment, without signs of local recurrence. When brain metastases occur, they usually present in the context of advanced disease and may mimic neurological events such as ischemic stroke.

Although current evidence is still limited to case reports, this experience supports the consideration of aggressive local treatment in combination with systemic therapy for patients with good performance status. Larger clinical trials are required to clarify the role of this approach in such rare malignancies. This case highlights the importance of long-term follow-up and a multidisciplinary approach in uncommon aggressive malignancies such as UA.
